# MSA-YOLO: A Remote Sensing Object Detection Model Based on Multi-Scale Strip Attention

**DOI:** 10.3390/s23156811

**Published:** 2023-07-30

**Authors:** Zihang Su, Jiong Yu, Haotian Tan, Xueqiang Wan, Kaiyang Qi

**Affiliations:** 1 School of Software, Xinjiang University, Urumqi 830091, China; yujiong@xju.edu.cn (J.Y.); wanxueqiang@stu.xju.edu.cn (X.W.); 107552101655@stu.xju.edu.cn (K.Q.); 2 College of Information Science and Engineering, Xinjiang University, Urumqi 830046, China; tanhaotian@stu.xju.edu.cn

**Keywords:** object detection, remote sensing images, attention mechanism, YOLO network

## Abstract

Remote sensing image object detection holds significant research value in resources and the environment. Nevertheless, complex background information and considerable size differences between objects in remote sensing images make it challenging. This paper proposes an efficient remote sensing image object detection model (MSA-YOLO) to improve detection performance. First, we propose a Multi-Scale Strip Convolution Attention Mechanism (MSCAM), which can reduce the introduction of background noise and fuse multi-scale features to enhance the focus of the model on foreground objects of various sizes. Second, we introduce the lightweight convolution module GSConv and propose an improved feature fusion layer, which makes the model more lightweight while improving detection accuracy. Finally, we propose the Wise-Focal CIoU loss function, which can reweight different samples to balance the contribution of different samples to the loss function, thereby improving the regression effect. Experimental results show that on the remote sensing image public datasets DIOR and HRRSD, the performance of our proposed MSA-YOLO model is significantly better than other existing methods.

## 1. Introduction

High-resolution remote sensing images are now more accessible with the continuous advancements in remote sensing and drone technology. Remote sensing image object detection is identifying and locating objects in high-resolution remote sensing images through remote sensing technology. It is a crucial task in remote sensing and satellite image analysis. The broad applicability and significant research value of remote sensing image object detection are evident in fields such as military reconnaissance, urban planning, and agricultural surveying [1,2].

Object detection includes traditional methods and deep learning-based methods. Although traditional detection methods show good interpretability, they require manual feature design, which might lead to sensitivity to changes in the shape and size of the object, making them difficult to adapt to complex scenes. Deep learning-based methods can automatically learn features from raw data, notably enhancing the accuracy and robustness of detection. Among deep learning-based methods, the algorithm based on Region Proposal appeared earliest, such as Faster R-CNN [3], Cascade R-CNN [4], and Mask R-CNN [5]. The detection task is partitioned into two stages in this type of algorithm. The Region Proposal Network (RPN) generates region proposals in the first stage, whereas the second stage performs object classification and location regression; although this two-stage detection algorithm can attain high detection results, its low detection speed can limit its applications in real-time detection scenarios. In contrast, a one-stage detection algorithm directly regresses and predicts each object without the Region Proposal stage, which leads to a much faster detection speed. RetinaNet [6], Single Shot MultiBox Detector (SSD) [7], and YOLO [8,9,10,11,12] series are representatives of one-stage detection algorithms. Meanwhile, anchor-free object detection methods without predefined anchors have become increasingly popular, with examples such as Fully Convolutional One-Stage Object Detection (FCOS) [13] and YOLOX [14]. Recently, object detectors based on transformers have attracted attention in object detection. DETR [15] is the first application of transformers in object detection, which does not require anchor boxes or region extraction networks, but directly predicts the position and category of objects from images using transformer networks. Deformable DETR [16] was improved on DETR, which uses deformable convolution to improve object feature extraction and position representation. Deformable DETR performs better than DETR in multiple object detection tasks. These object detection methods based on deep learning have gained widespread use across various natural-scene object detection tasks, where they have demonstrated excellent performance.

**Figure 1:**
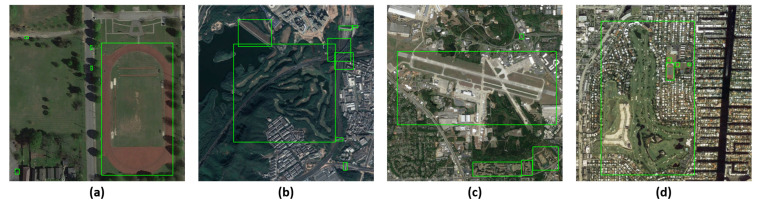
Image samples in the DIOR dataset: (**a**,**b**) show the problem of significant differences in object size; (**c**,**d**) show the complexity of background noise.

Remote sensing image object detection presents a significant challenge due to pronounced dissimilarities between remote sensing images and natural images. Specifically, natural images are typically taken horizontally from the ground, whereas remote sensing images are captured from a high altitude and are perpendicular to the ground. The difference in imaging angles will induce variations in the characteristics of the same kind of objects. In addition, remote sensing images offer a vast field of view, often resulting in the inclusion of many complex background information. The background noise will interfere with the recognition and extraction of object features, negatively impacting detection outcomes. Further, the size of different objects in remote sensing images varies greatly, and the size of the same object will also change according to the shooting height. The drastic size differences and variations can lead to degradation of detection performance. Figure 1 illustrates remote sensing image samples that exemplify differences between remote sensing and natural images. Consequently, the detection performance of the generic object detection methods will decrease when they are directly applied to the remote sensing image object detection task.

To address the abovementioned issues, we propose an efficient remote sensing image object detection model, MSA-YOLO, based on the YOLOv5s baseline model in this article. Our method simultaneously considers both detection accuracy and a lightweight model. The main contributions of this article are summarized as follows:

- We propose a multi-scale attention mechanism based on a strip convolution to solve the problems of complex background noise and significant object scale differences in object detection of remote sensing images. We add this attention module to the baseline model to reduce the effect of background noise and fuse multi-scale features.
- We introduce GSConv into the feature fusion layer of the model and propose a new GS-PANet. It can improve the detection accuracy and reduce the number of parameters of the model, making the model more applicable to practical remote sensing image detection tasks.
- We optimize the original loss function in YOLOv5 and propose a new Wise-Focal CIoU loss function. It allows the model to better balance the contribution of the loss function to different samples during training and thus optimize the regression performance of the model.
- We conducted multiple experiments on large-scale public datasets of remote sensing images. The results show that our MSA-YOLO model performs well in object detection of remote sensing images, proving the effectiveness and feasibility of our methods proposed in this article.

The structure of the rest of this article is as follows. Section 2 briefly introduces related work. Section 3 details the specific content of our proposed method. Section 4 presents experimental results and analysis. Finally, Section 5 summarizes the article.

## 2. Related Work

### 2.1. Object Detection for Remote Sensing Images

Before deep learning was applied to object detection, traditional remote sensing image object detection methods mainly included threshold-based clustering, template matching, and feature extraction. The authors of [17] proposed a method for object detection based on mean shift segmentation and non-parametric clustering, which uses prior knowledge of the object shape and a hierarchical clustering method for object extraction and clustering. The authors of [18] proposed a remote sensing image object detection method based on feature extraction, which proposes an improved SIFT (Scale-Invariant Feature Transform) algorithm to extract uniformly distributed matching features, then refines the initial matching by binary histogram and random sample consensus. Traditional remote sensing image detection methods usually require prior knowledge to design features manually, which are then poorly robust to complex scene changes and noise disturbances, resulting in low detection performance.

With the widespread application of deep learning in object detection, many researchers have improved the general detectors applied to remote sensing image object detection tasks. The authors of [19] proposed a contextual refinement module for remote sensing images based on Faster R-CNN to extract and refine the contextual information and improve the Region Proposal Network (RPN) to obtain more positive samples. However, it did not consider the problem of background noise. L-SNR-YOLO [20] constructs a network backbone by a swin-transformer and convolutional neural network (CNN) to obtain multi-scale global and local information. Moreover, a feature enhancement module is proposed to make image features salient. However, this approach should have considered the lightweight of the model, which leads to the introduction of a large number of parameters. LOCO [21] proposes a variant of YOLO that uses the spatial characteristics of the object to design the layer structure of the model and uses constrained regression modeling to improve the robustness of the predictions, which allows for better detection of small and dense building footprints. TPH-YOLOv5 [22] adds a detection layer for small objects based on YOLOv5 and introduces a spatial attention mechanism and transformer encoder module, significantly improving the detection accuracy for small objects in UAV images, but it neglected the large variations of object scale in remote sensing images. DFPH-YOLO [23] proposes a dense feature pyramid network for remote sensing images based on YOLOv3, which enables four detection layers to combine semantic information before and after sampling to improve object detection performance at different scales. However, the introduction of irrelevant background information was not avoided.

Recently, some studies have proposed new strategies and methods for remote sensing image object detection. LAG [24] proposes a hierarchical anchor generation algorithm that generates anchors in different layers based on the diagonal and aspect ratio of the object, making the anchors in each layer match better with the detection range of that layer. The authors of [25] proposed a new multi-scale deformable attention module and a multi-level feature aggregation module and inserts them into the feature pyramid network (FPN) to improve the detection performance of various shapes and sizes of remote sensing objects. RSADet [26] considers the spatial distribution, scale, and orientation changes of the objects in remote sensing images by introducing deformable convolution and a new bounding box confidence prediction branch. The authors of [27] proposed to cast the bounding box regression in the aerial images as a center probability map prediction problem, thus largely eliminating the ambiguities on the object definitions and the background pixels. Although these above studies provide optimization schemes for remote sensing image detection, they neglect the problem of background noise introduction when the model performs feature extraction on elongated objects in remote sensing images. In addition, the problem of weight allocation of different quality samples in images to the regression process and the lightweight design of the model also need to be considered.

### 2.2. Attention Mechanism

Currently, attention mechanisms have been widely applied in the field of image processing. The attention mechanisms can adaptively select essential parts the network should focus on, thereby improving its feature extraction ability. The attention mechanisms can be divided into spatial and channel attention mechanisms. The spatial attention mechanism guides the model to focus on critical spatial regions in a weighted manner, thereby improving the perception ability of the network for image details. The channel attention mechanism learns the weights of each channel, allowing the network to pay more attention to the critical channels in the image during the training process, thereby improving the ability of the network to extract image features.

The Squeeze-and-Excitation Network (SENet) [28] is a classic channel attention method. It first compresses the features on the channel by global average pooling, then learns the weight of each channel through two fully connected layers, thus weighting the channel importance of the input feature map to learn the relationship between global channel information. Efficient Channel Attention (ECA) [29] improves on SENet by using one-dimensional convolution with adaptive convolution kernel size instead of global connections, learning more practical information in a more efficient way but ignoring the relationship between global channel information. The Convolutional Block Attention Module (CBAM) [30] is a mixed attention method in both channel and spatial domains, which combines channel and spatial attention by performing average pooling and max pooling operations on the input feature map. Coordinate Attention (CA) [31] is a spatial position-based attention mechanism. It extracts information through horizontal and vertical direction average pooling operations, encodes the spatial position information of the input feature map into two-dimensional coordinates, fuses coordinate information into channel information, and then pays attention, and is a very effective attention mechanism. Triplet Attention Module (TAM) [32] is a rotation attention mechanism that rotates the feature map so that the model can focus on different parts of the object in different directions, thereby improving the accuracy of object detection and image classification. Non-Local [33], Self-Calibrated Convolutions (SC) [34], and Bi-Level Routing Attention (BRA) [35] are all self-attention mechanisms used for computer vision tasks. This method establishes a relationship between pixels in the image and weights them semantically, but often introduces a large number of additional parameters.

These attention mechanisms perform well in images in natural scenes by adaptively calibrating and directing the network to focus more on the foreground of the feature map, thus slightly mitigating the interference of background noise in the image. However, for remote sensing images, none of the above attention methods adopts an effective strategy to reduce the introduction of background noise and ignores the problem of scale differences of remote sensing objects. Hence, the performance improvement in the object detection task of remote sensing images is lower than in natural scenes.

### 2.3. GSConv

**Figure 2:**
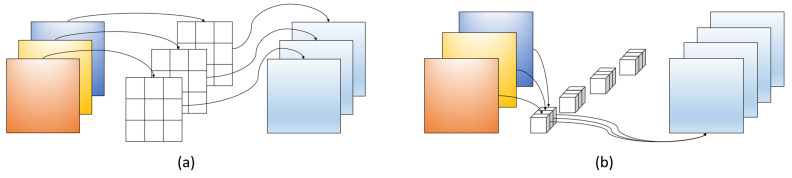
Illustration of two types of convolution: (**a**) Depthwise Convolution; (**b**) Pointwise Convolution.

In order to improve the performance and efficiency of networks, the study of lightweight models has also received widespread attention. Models such as MobileNet [36], ShuffleNet [37], and EfficientNet [38] achieve lightweight design through different techniques. Among them, Depthwise Separable Convolution is a common lightweight convolution technique consisting of two steps: Depthwise Convolution (DWConv) and Pointwise Convolution (PWConv). Figure 2a shows the framework of DWConv. It applies a separate convolution kernel to each channel of the input tensor, for example, using C convolution kernels to perform convolution operations on an input tensor with C channels. The size of each convolution kernel is usually small, such as 3 × 3 or 5 × 5. PWConv, as shown in Figure 2b, applies a 1 × 1 convolution kernel for dense calculation, which can fuse information between channels and reduce dimensionality.

DWConv is equivalent to a grouped convolution with the number of groups equal to the number of channels in the input tensor. Each channel is calculated using a separate convolution kernel. Although this method can significantly reduce the number of parameters and computation costs, there is no interaction between channels, and the information between channels is separated during the calculation. It is an important reason for the low accuracy of DWConv calculation. PWConv fuses channel information through dense convolution operations of 1 × 1, which has higher calculation accuracy. However, this dense convolution also brings more parameters and computational costs. Depthwise Separable Convolution uses DWConv and PWConv but simply connects them in series, and the dense calculation results between channels are separated. Compared with ordinary convolution, although the number of parameters is reduced, the calculation accuracy is lower, which will affect the detection performance of the model.

**Figure 3:**
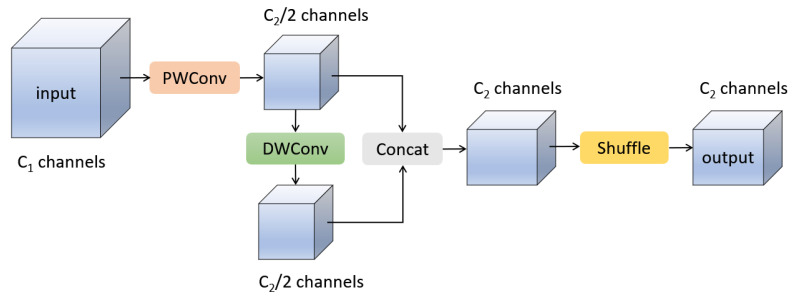
Structure of GSConv module.

To solve the abovementioned problem, Li et al. proposed GSConv [39], a new lightweight convolution technique. The structure of the GSConv module is shown in Figure 3, where PWConv and DWConv represent Pointwise Convolution and Depthwise Convolution in depth-separable convolution, respectively. They are combined in a more efficient way in GSConv. Assuming that the number of channels in the input tensor is C1 and the number of channels in the output tensor is C2, first, to obtain more accurate dense calculation results, the module uses a PWConv to calculate the input tensor and compresses the channels to 1/2 of the output channels. Then, to ensure lightweight computation, a depth-wise DWConv operation is performed on the dense computation result of PWConv to obtain a result with C2/2 channels. These two calculation results obtained above are then stacked along the channel dimension to obtain a tensor with C2 channels. Finally, to mix the calculation results of PWConv and DWConv, a shuffle operation is applied along the channel dimension, allowing the information generated by PWConv to permeate into different parts of the computation result of DWConv. GSConv combines the accuracy of dense computation and the lightweight characteristics of depth-wise computation, making it an efficient, lightweight convolution method.

## 3. Methods

### 3.1. Review of YOLOv5s

**Figure 4:**
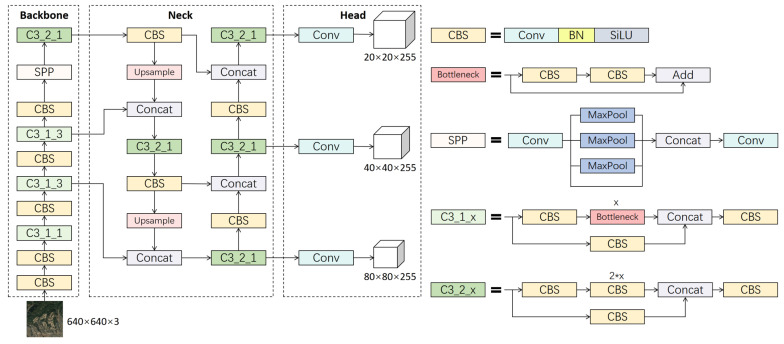
Overall structure of YOLOv5s model.

YOLOv5 is a one-stage object detection algorithm developed by Ultralytics. It has multiple versions based on model size, including n, s, m, l, and x. YOLOv5s is a relatively lightweight model suitable for deployment on lightweight devices. The overall network structure of YOLOv5s is shown in Figure 4. It consists of four parts: input, backbone, neck, and head. The default input image size of the input part is 640 × 640. Multiple data augmentation strategies are adopted to increase the diversity of the training data, thereby improving the robustness and generalization ability of the model. The backbone of YOLOv5s uses CSPDarknet53, which consists of C3 modules and SPP (Spatial Pyramid Pooling) modules. The C3 module adopts the CSP (Cross Stage Partial) structure, which separates the information flow of the network into two branches for processing. The feature tensor of the main branch is divided into two parts. One part is concatenated with the output of the branch after the convolutional operation and residual connection, whereas the other part is directly concatenated with the output of the branch. This design can prevent the network from losing too much information during information transmission, thus improving the utilization of features. In addition, the residual connection can effectively prevent the gradient disappearance problem in deep neural networks. The SPP module is a pooling operation that processes the feature maps of the backbone with different sizes of pooling kernels to fuse spatial information of different receptive field sizes. The primary function of the SPP module is to improve the ability of the model to detect objects of various scales without changing the input size. The neck part of the YOLOv5s network uses the PANet (Path Aggregation Network) structure, which can effectively fuse feature maps of different scales to improve detection accuracy. PANet consists of two parts: FPN (Feature Pyramid Network) [40] and PAN. FPN is used to generate feature pyramids of different scales, and PAN is used to fuse these feature pyramids. PAN not only performs feature fusion by upsampling the feature maps but also performs another round of feature fusion by downsampling to enrich semantic information, which is beneficial for detecting objects of multiple scales. The head of YOLOv5s adopts the structure of YOLOv3, including three output layers of different scales. Each output layer is responsible for classifying and bounding box regression of feature maps of different sizes to obtain the final detection results.

The YOLOv5s model has gained popularity across different industries like healthcare, transportation, and manufacturing. However, there is a significant difference between remote sensing images and natural images, and YOLOv5s does not perform well in object detection tasks on remote sensing images. This article takes YOLOv5s as the baseline model and improves in multiple aspects to make it better suited for remote sensing image object detection tasks.

### 3.2. Multi-Scale Strip Convolution Attention Mechanism

**Figure 5:**
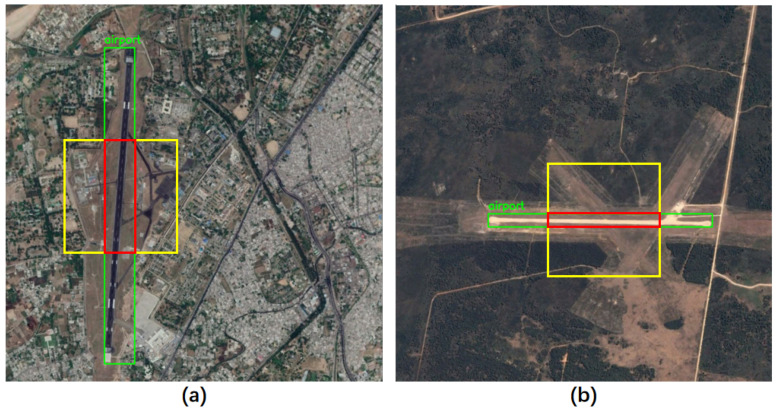
Illustration of using strip convolutions for strip-shaped objects in remote sensing images: (**a**) Vertical strip convolution; (**b**) Horizontal strip convolution. The green box represents the ground truth label, the yellow box represents the standard convolution, and the red box represents the strip convolution.

The extraction and recognition of foreground features can be hindered by complex background noise, which is an important reason for the poor performance of remote sensing image object detection. The attention mechanism can improve the focus of the model on foreground objects in the feature map, thereby reducing the influence of background noise to a certain extent. Through the observation of remote sensing image data, we found that some objects, like airports, dams, and bridges, may appear as narrow stripes due to the specific shooting height and angle. As shown in Figure 5, when using a regular square convolution to extract features of the long strip-shaped airport, the surrounding background information of the object will inevitably be fused with the object features, resulting in a large amount of background noise and reducing detection performance. In addition, the problem of significant differences in object scales in remote sensing images will seriously reduce the performance of the detector, as the detector cannot simultaneously detect objects with significant scale differences.

In computer vision tasks, strip convolution can cover long strip-shaped object areas, capture long-range contextual information in a single direction, and avoid introducing additional background noise. Therefore, based on the above content, we propose a multiscale strip convolution attention mechanism called MSCAM. This method uses strip convolutions of different directions to focus on the horizontal and vertical information in the feature map. It uses convolutions of different sizes to fuse features of different scales. This method can simultaneously solve the problems of complex background noise and significant differences in object scales in remote sensing images. Next, we will provide a detailed introduction to the implementation details of this method.

First, we split the input feature map into two sub-feature maps in the channel dimension to perform different operations on the two while reducing the parameters. Then, to avoid the problem of introducing additional background noise with square convolution, we use 1 × n and n × 1 strip convolutions for the two sub-feature maps, respectively, to focus on spatial information in the horizontal and vertical directions of the feature map. Furthermore, to address the problem of object scale differences in remote sensing images, we use three different convolution kernel sizes for each sub-feature map of strip convolutions. This method allows us to focus on multiple different scales of objects and fuse multi-scale features.

Specifically, we split X evenly into two parts in the channel dimension for an input feature map X with C channels, as shown in Equation (1):

(1) $$X_{i\in [1,2]}=X_{s}\left[c*\left(i-1\right),c*i\right]$$

where $X_{s}$ denotes the split operation applied to the input feature map *X*, where c=C/2 means *X* is equally divided into two sub-feature maps, each having half the number of channels as the original input feature map. $X_{i}$ represents the sub-feature map obtained after the split operation.

Next, we perform operations on the two sub-feature maps obtained from the split operation. Both sub-feature maps *A* and *B* undergo three parallel strip convolutions with different kernel sizes, focusing on spatial information in the weight and height directions of the feature map. Then, the results of the three convolutions are added and undergo nonlinear transformation through an activation function, which can be represented as Equations (2) and (3):

(2) $$Y_{A}=S\left(\sum_{i=1}^{3}F\left(A,K_{i}\right)\right)$$

(3) $$Y_{B}=S\left(\sum_{i=1}^{3}G\left(B,K_{i}\right)\right)$$

where *F* represents the convolution of feature map *A* with a strip-shaped kernel of size $1\;\times \;K_{i}$, and *G* represents the convolution of feature map *B* with a strip-shaped kernel of size $K_{i}\;\times \;1$. *S* means the SiLU activation function and $Y_{A}$ and $Y_{B}$ represent the feature maps obtained by adding the convolution results from different sizes of kernels. We use strip convolution for two reasons. First, it allows for extracting horizontal and vertical information separately from different directions, avoiding introducing a large amount of background noise that square convolution would cause. Second, a strip convolution is relatively lightweight compared to a standard convolution. $K_{i}$ represents the size of the convolution kernel, which affects the receptive field of a feature map and significantly impacts detection performance. Using convolution kernels of varying sizes is vital to computing features at different scales. To determine the kernel size for each strip convolution, we propose a mapping function represented by Equation (4).

(4) $$K_{i\in [1,2,3]}=2\left\lfloor \frac{\min(w,h)}{2^{i}+1}\right\rfloor +1$$

In Equation (4), *w* and *h* represent the width and height of the input feature map, ⌊⌋ represents the floor operation, and $K_{i}$ represents the size of the strip convolution kernel. Equation (4) can calculate three different sizes of convolution kernels according to the size of the input feature map. Using three strip convolutions with different kernel sizes can extract multi-scale features, thereby focusing on objects of different scales in the image and solving the problem of significant differences in object scales in remote sensing images.

Afterward, $Y_{A}$ and $Y_{B}$ are concatenated along the channel dimension to obtain a feature map with *C* channels. Then, a 1 × 1 convolution is employed to simulate the relationship between different channels. The convolution result is used as attention weights and multiplied with the original input, thereby re-balancing the input of the module, as shown in Equation (5):

(5) $$\;\mathrm{Out}\;=f\left(\mathrm{Concat}\left(Y_{A},Y_{B}\right)\right)*X$$

where *f* represents a convolution operation of size 1 × 1, Concat represents a concatenation operation along the channel dimension, and ∗ represents the dot product between the attention weights and the input feature map *X*, which applies attention weights to the original input feature map. Out represents the output feature map filtered by the attention weights.

**Figure 6:**
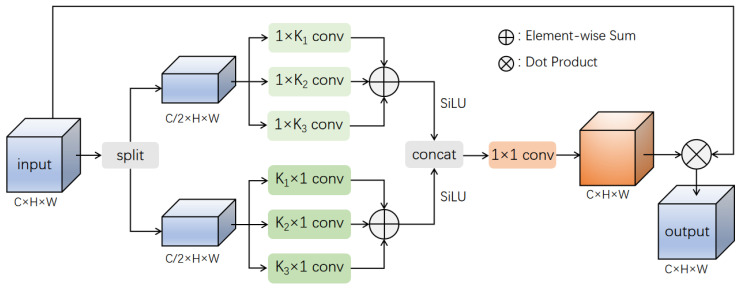
Structure of MSCAM.

**Figure 7:**
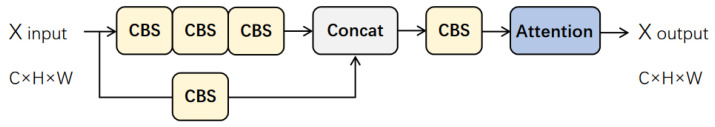
Diagram of integrating MSCAM into backbone.

Integration strategy: The MSCAM proposed in this paper is a plug-and-play module, and its overall structure is shown in Figure 6. In the experimental part, we applied the attention module to the last convolutional block of the network backbone. In the comparative experiment, other attention modules were added to the same position, as shown in Figure 7.

### 3.3. Improved PANet by GSConv

In practical applications of remote sensing image object detection tasks, models often need to be deployed and run in resource-limited environments, such as drones and satellites. Therefore, we need to adopt a lightweight design strategy for remote sensing image object detection models to reduce the size and complexity of the models, making them more suitable for practical application scenarios. In the YOLOv5s network architecture, the feature fusion part adopts the Path Aggregation Network (PANet) structure, which performs another bottom-up fusion based on the top-down feature fusion of the Feature Pyramid Network (FPN), enabling shallow information to be more utilized in deep layers of the network. However, this approach introduces a longer propagation path and more convolutional operations, resulting in an increase in the number of model parameters, which is not conducive to model lightweighting.

In the feature fusion part of the network, the feature map usually contains numerous channels but has smaller width and height dimensions. This elongated tensor shape is suitable for lightweight processing with DWConv, but the sparse calculation of DWConv can lead to a decrease in accuracy. The dense calculation of PWConv can avoid the loss of semantic information caused by sparse connections. GSConv combines the calculation results of PWConv and DWConv through shuffle, mixing the precise results of PWConv dense calculation into the calculation results of DWConv, thus achieving a lightweight and efficient convolution method. Therefore, in this work, we introduce GSConv into the feature fusion part of the network to achieve a new lightweight feature fusion network called GS-PANet.

**Figure 8:**
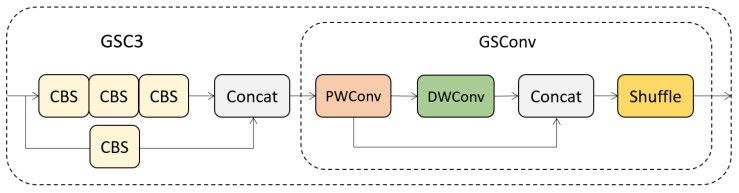
Structure of GSC3 module.

First, based on the superiority of GSConv, we replace the ordinary convolution in the original PANet with a GSConv module. Then, by introducing GSConv to improve the C3 module, we design a more efficient GSC3 module, whose structure is shown in Figure 8. The input feature map first undergoes feature extraction in two parts through the CSP structure, and then the merged result is input into GSConv for mixing the results of PWConv and DWConv.

**Figure 9:**
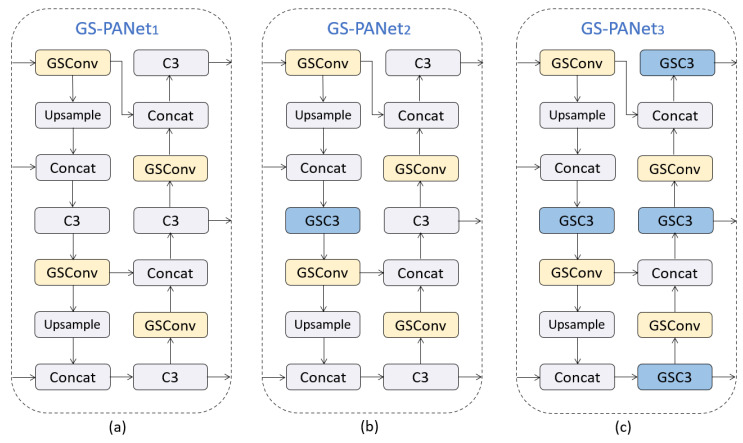
Three design schemes of GS-PANet: (**a**) GS-PANet1; (**b**) GS-PANet2; (**c**) GS-PANet3.

The GSC3 module can replace any C3 module in the feature fusion layer of the network. To explore the best embedding scheme for the GSC3 module, we provide three design schemes for the GS-PANet, whose structures are shown in Figure 9. Among them, GS-PANet1 only introduces GSConv, GS-PANet2 replaces the first C3 module in the original PANet with the GSC3 module while introducing GSConv, and GS-PANet3 replaces all C3 modules with GSC3 modules. Table 1 shows the comparative experimental results of the three structures of GS-PANet1, 2, and 3. It can be seen that although the GS-PANet3 method, which uses more GSC3 modules, reduces the parameter volume more, it also loses some detection accuracy. We analyze that because the three additional GSC3 modules of GS-PANet3 are closer to the detection head than GS-PANet2, this convolutional layer often needs a larger receptive field to focus on the overall features of the object, so it is not suitable to use a lighter GSC3 module. Therefore, we adopt the more excellent performance of GS-PANet2 as the final design scheme.

### 3.4. Wise-Focal CIoU Loss

The loss function in object detection is an indicator used to measure the difference between the predicted results of the model output and the ground–truth labels. By minimizing the loss function, the model is optimized to make its output closer to the ground–truth labels. Therefore, designing a suitable loss function has a crucial influence on the final detection effect of the model. In the YOLOv5 algorithm, the loss function consists of three parts: confidence loss, classification loss, and localization loss. Among them, the localization loss has a direct impact on the localization effect of the predicted box. The YOLOv5 utilizes the CIoU loss function for localization to guide the bounding box regression process. It considers three aspects: the overlap between the predicted and ground–truth boxes, the distance between their center points, and the aspect ratio. The calculation process for CIoU loss is shown in Equations (6) and (7).

(6) $$L_{CIoU}=1-IoU+\frac{\rho^{2}\left(b,b^{gt}\right)}{c^{2}}+\left(\frac{v}{(1-IoU)+v}\right)v$$

(7) $$v=\frac{4}{\pi^{2}}{\left(\arctan\frac{w^{gt}}{h^{gt}}-\arctan\frac{w}{h}\right)}^{2}$$

where IoU represents the intersection over the union of the predicted box and the ground–truth box area, *b* and $b^{gt}$ represent the center point coordinates of the predicted box and the ground–truth box, respectively, ρ(∗) represents the Euclidean distance, *c* represents the diagonal distance of the minimum bounding rectangle of the predicted box and the ground–truth box, and *v* is the weight function used to measure the similarity of the aspect ratio.

In remote sensing image object detection, due to the complexity of the image background and the differences in object size and shape, the same image usually contains samples of different qualities, and the importance of varying quality samples to the regression process is different. Since the model only performs the bounding box regression process on the positive samples containing the foreground objects, we only focus on the effect of the quality of the positive samples here. For positive samples, the higher the IoU, the higher the quality of the sample. High-quality samples with high IoU provide more accurate and clearer object information, which can guide the training of the model more accurately than low-quality samples. This conclusion has been demonstrated in Prime Sample Attention (PISA) [41]. Therefore, for the same consideration, we expect high-quality samples to be given more weight than low-quality samples in remote sensing images.

**Figure 10:**
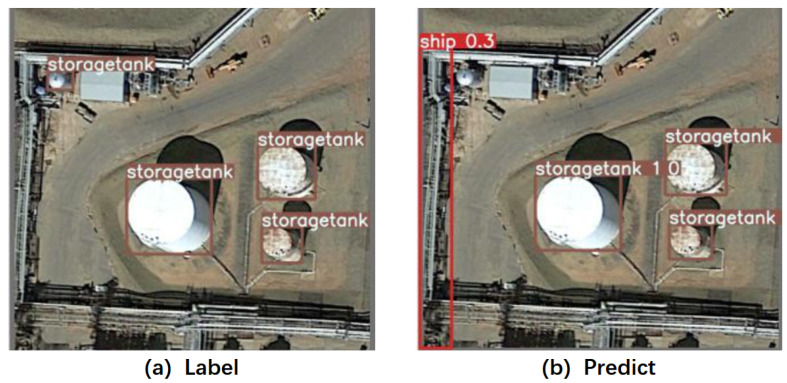
(**a**) Ground–truth label; (**b**) result of regression prediction using CIoU loss.

Through the study of CIoU loss, we found that although it considers the IoU, center point distance, and aspect ratio between the predicted box and the ground–truth box, it does not consider the difference in the importance of different quality samples to the regression process. CIoU loss optimizes samples of different qualities with equal weights, which is unfair to more important high-quality samples. At the same time, the low-quality samples with low IoU have too much weight, which will interfere with the regression of high-quality samples with high IoU, leading to a decrease in regression accuracy. As shown in Figure 10, the result shows that the network mistakenly predicted a ship object with a confidence of only 0.3 and interfered with the regression of other samples, resulting in a missed detection of a storage tank at the same location.

Libra R-CNN [42] and Dynamic R-CNN [43] propose that high-quality samples should have more gradient contribution in the model optimization process. They revised the SmoothL1 loss [44] to reweight these predicted bounding boxes. Therefore, the CIoU loss also needs to be optimized to address this issue. We first introduce a power transformation γ greater than 1 for the regression penalty term of CIoU loss, thereby enhancing the weight contribution of high-quality samples in regression to enhance their impact on model training. After introducing the power transformation, we propose Wise CIoU loss, whose calculation process is shown in Equation (8).

(8) $$L_{\mathrm{Wise}\;\mathrm{CIoU}\;}=1-IoU+\frac{\rho^{2\gamma }\left(b,b^{gt}\right)}{c^{2}}+{\left(\frac{v}{(1-IoU)+v}\right)}^{\gamma }v^{\gamma }$$

The Wise CIoU loss introduces a power transformation γ in the Euclidean distance between the predicted box, the ground truth box, and the aspect ratio regression term. Since γ is greater than 1, the Wise CIoU loss further amplifies the penalty terms of the Euclidean distance and aspect ratio between the predicted box and the ground truth box to amplify the weights of high-quality samples in the training process. It can adjust the weight of the loss function more effectively according to the current sample and increase the loss function contributions of high-quality samples. In addition, since the model pays more attention to high-quality samples with high IoU, it can also alleviate the interference of complex background noise in remote sensing images. γ is a hyperparameter in the training process, and we will discuss its value later.

The Wise CIoU loss increases the loss weight of high-quality samples with high IoU. However, in remote sensing image object detection, the vast majority of predicted boxes obtained based on anchors have small IoU between them and the ground truth boxes, implying the existence of a large number of low-quality samples (outliers). EIoU loss [45] suggests that these large number of low-quality samples with low IoU tend to cause drastic fluctuations in the regression loss of training. Therefore, to further reduce the negative impact of low-quality samples in remote sensing images on the regression loss, we improve based on Wise CIoU loss. Inspired by the EIoU loss, we propose a new loss function Wise-Focal CIoU loss, which is calculated as shown in Equation (9).

(9) $$L_{\mathrm{Wise}-\mathrm{Focal}\;\mathrm{CIoU}\;}=IoU^{\beta }\left(1-IoU+\frac{\rho^{2\gamma }\left(b,b^{gt}\right)}{c^{2}}+{\left(\frac{v}{(1-IoU)+v}\right)}^{\gamma }v^{\gamma }\right)$$

Specifically, we multiply Wise CIoU loss by a balance term $IoU^{\beta }$, which is used to suppress the weight of low-quality samples to obtain the final form of Wise-Focal CIoU loss. $IoU^{\beta }$ reweights Wise CIoU loss, where β is a hyperparameter that controls the extent of outlier suppression, and we keep the same value as in EIoU loss with $IoU^{\beta }=0.5$. The introduction of the balance term $IoU^{\beta }$ can suppress the gradient contribution of low-quality samples with low IoU in the regression loss, avoiding a large number of low-quality samples in remote sensing images from dominating the loss leading to a skewed loss function, thereby further enhancing the effect of model regression.

In summary, our proposed Wise-Focal CIoU loss provides a reasonable re-weighting of samples of different quality in the regression process by introducing a power transformation and a balance term. In this way, the contribution of samples of different quality to the regression loss can be flexibly adjusted, thus effectively improving the accuracy and robustness of remote sensing image object detection.

Hyperparameter discussion: a power transformation γ>1 to be introduced in the Wise-Focal CIoU. To explore the effect of different values of γ, we conducted a series of comparative experiments, with γ set to 2, 3, 4, 5, and 6, respectively. The experimental results are shown in Table 2.

The experimental results in Table 2 show that the detection accuracy gradually decreases as the power transformation γ increases, and the best result is obtained when γ is set to 2. In the subsequent experiments, we will uniformly set the value of γ to 2.

### 3.5. Discussion

**Figure 11:**
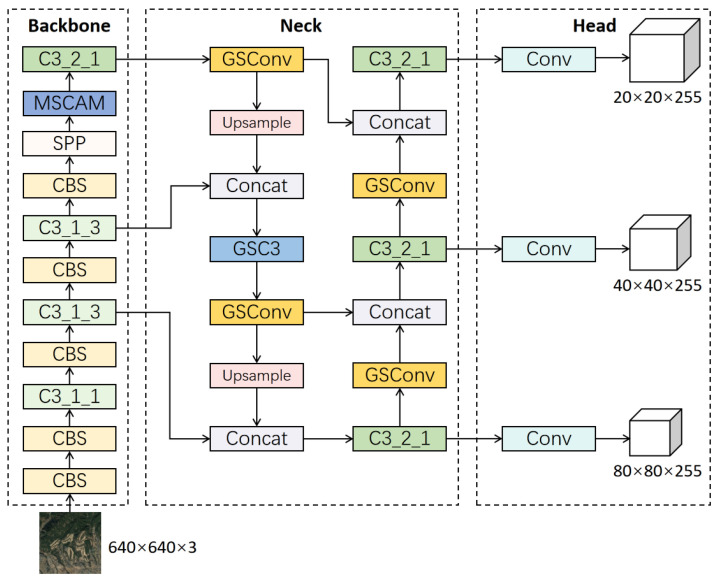
Overall structure of MSA-YOLO model.

The overall network structure of our proposed MSA-YOLO model is shown in Figure 11. Our MSCAM is integrated into the backbone of the network to minimize the effect of a large amount of background noise in the remote sensing images. This challenge of different background landscapes also relates to spatial heterogeneity. A spatial transformation module to address the spatial heterogeneity problem is proposed in STN [46], which can solve the problem by generating appropriate transformations for each input, and then performing multiple spatial transformations, including scaling, cropping, and rotating on the whole feature map. This approach allows the network to learn more spatial invariance. However, the problem that needs to be addressed in remote sensing images is the irrelevant background noise that is inevitably introduced when feature extraction is performed on strip-shaped objects. Therefore, we chose to use an attention mechanism based on strip convolutions to minimize the effect of extraneous background noise instead of using spatial transformation.

## 4. Experiments

### 4.1. Datasets

**Figure 12:**
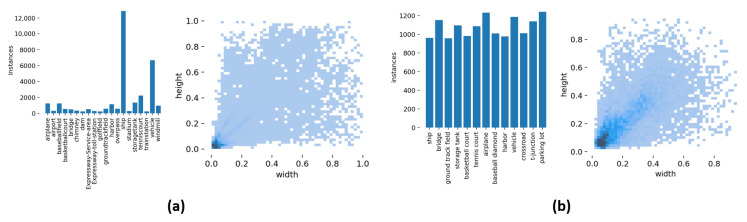
Data distribution in two datasets: (**a**) DIOR; (**b**) HRRSD.

For our experiments, we used two extensive remote sensing datasets that are publicly available: DIOR [2] and HRRSD [47]. The DIOR dataset consists of 23,463 images and 192,472 objects containing 20 object categories. The HRRSD dataset contains 21,761 images and 55,740 objects covering 13 different object categories. The images in both datasets exhibit rich variations in weather, lighting, and background, and the object scales are diverse, which can well reflect the characteristics of the complexity of remote sensing image backgrounds and object size differences. Figure 12 shows the number of samples and the distribution of object sizes for all categories in the DIOR dataset and HRRSD dataset. It can be seen that there is an obvious category imbalance problem in the DIOR dataset, and the objects in both datasets have a significant size difference.

### 4.2. Experiments Settings

Our experiments are based on the PyTorch 1.7.1 framework and use an NVIDIA GeForce RTX 2080Ti GPU. The input image size is 800 × 800 and the initial learning rate is 0.01. We use the stochastic gradient descent (SGD) optimizer with a momentum of 0.937 and a weight decay coefficient of $5\times 10^{-4}$. We start training from three warm-up epochs and perform 200 epochs and the batch size is 16. In all experiments, some data augmentation methods are adopted in the training phase, including random HSV transformation, random translation, random scaling, and mosaic strategy. All experiments are conducted under the same experimental environment and parameter settings.

### 4.3. Evaluation Metrics

The basic evaluation indicators in object detection include Precision, Recall, and AP. Precision indicates the proportion of true positive samples in all samples classified as positive. Recall indicates the proportion of samples correctly identified as positive in all true positive samples. Precision indicates the detection accuracy of the model, whereas Recall indicates the detection completeness of the model. These two indicators often conflict with each other. AP is an integrated consideration of Precision and Recall, which can reflect the comprehensive performance of the model in identifying a specific category. It is a relatively comprehensive accuracy evaluation indicator.

In this article, we use evaluation metrics proposed by MSCOCO [48] to assess the detection accuracy of the model, including $AP_{50}$, $AP_{75}$, $AP_{50:95}$, $AP_{S}$, $AP_{M}$, and $AP_{L}$. Among them, $AP_{50}$ and $AP_{75}$ represent the average AP value of all categories when the IoU threshold is set to 0.5 and 0.75. $AP_{50:95}$ is the average AP value of all categories when the IoU threshold ranges from 0.5 to 0.95 with a step length of 0.05. It is a more rigorous and reflective detection metric of the actual performance of the model. $AP_{S}$, $AP_{M}$, and $AP_{L}$ correspond to the average AP of small, medium, and large objects. In addition, the number of parameters in the model determines the size of the model, so we leverage this metric to assess the practical feasibility of model deployment.

### 4.4. Experiment Results and Discussion

#### 4.4.1. Ablation Experiments

In order to verify the effectiveness of each improvement module in our proposed method, we conducted ablation experiments on the DIOR dataset. MSCAM, GS-PANet, and Wise-Focal CIoU loss were sequentially added to the baseline model, and the experimental results are shown in Table 3.

After incorporating our proposed MSCAM into the network backbone, AP50 and AP50:95 improved by 1.9% and 3.6%, respectively, with only a 0.27 M increase in model parameters. It indicates that our attention mechanism can effectively improve detection accuracy, and using channel grouping and lightweight strip convolutions avoids the dramatic increase in the number of parameters caused by convolutions with a large kernel. The introduction of GS-PANet not only improved the detection accuracy of the model but also made it more lightweight, demonstrating that GS-PANet indeed combines the advantages of Pointwise and Depthwise Convolutions after the introduction of GSConv. After replacing the original CIoU loss with Wise-Focal CIoU loss, AP50 decreased slightly by 0.39%, but the more stringent AP50:95 metric improved by 0.9%. After analysis, we believe that Wise-Focal CIoU loss increases the weight of high-quality samples with high IoU while decreasing the weight of low-quality samples with low IoU, leading to a decrease in the AP metric under low IoU thresholds. When applying GSConv and the Wise-Focal CIoU loss function to the model simultaneously, AP50:95 improved significantly by 2.7%. Finally, we integrated all of our methods into the baseline model to create the MSA-YOLO model proposed in this study. With fewer model parameters than the baseline YOLOv5s, MSA-YOLO achieved a 1.9% and 5.2% improvement in AP50 and AP50:95, respectively, demonstrating better detection performance.

#### 4.4.2. Comparative Experiments

To verify the superiority of the attention mechanism proposed in this study, we conducted comparative experiments on the DIOR dataset. In the experiment, we added all attention modules at the same location in the model backbone. As shown in Table 4, MSCAM achieves the best detection accuracy compared to other attention methods. MSCAM effectively improves detection accuracy with only a small increase in parameters. Among them, the detection accuracy of medium and large objects has dramatically improved, but the detection of small objects only shows a slight improvement. We believe that there are two main reasons for this phenomenon. First, the size of the convolution kernels used in our MSCAM is large. The larger convolution kernel can cover a more extensive spatial range, which is beneficial for detecting large objects. However, the larger receptive field can also lead to the loss of small features and affect the detection of small objects. Second, there is no significant aspect ratio difference in small objects, so MSCAM with striped convolution does not improve the detection effect of small objects. Nevertheless, overall, the attention method proposed in this study still achieved competitive results.

**Figure 13:**
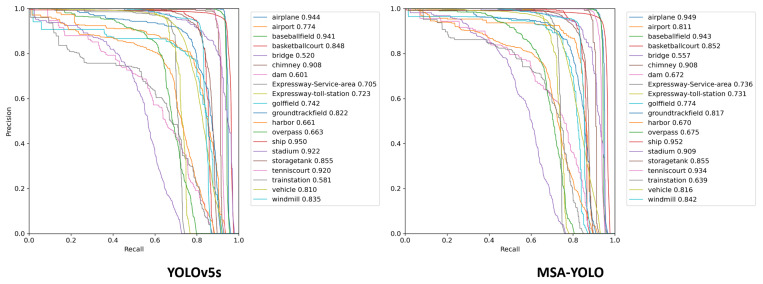
Precision–Recall curves and AP values of YOLOv5s and MSA-YOLO on the DIOR dataset.

To verify the effectiveness of our proposed model, we compared MSA-YOLO with the baseline model YOLOv5s. Figure 13 shows the Precision–Recall curves and AP values of YOLOv5s and MSA-YOLO on each category of the DIOR dataset when the IoU threshold is 0.5. It can be seen that MSA-YOLO performs better than YOLOv5s in the detection of the 18 categories, such as Airplane, Airport, Vehicle, and Windmill. The improvement is particularly significant in some elongated objects that are difficult to detect. For example, the AP of MSA-YOLO in Bridges, Dam, and Train Station significantly increased by 7.1%, 11.8%, and 9.9%, respectively. As for the Ground Track Field and Stadium categories, MSA-YOLO reduced the AP by 0.6% and 1.4%, respectively, compared to YOLOv5s. We argue that these types of objects have a relatively regular shape and large size, and the strip convolution cannot effectively capture their global semantic information, resulting in a slight decrease in detection performance.

**Figure 14:**
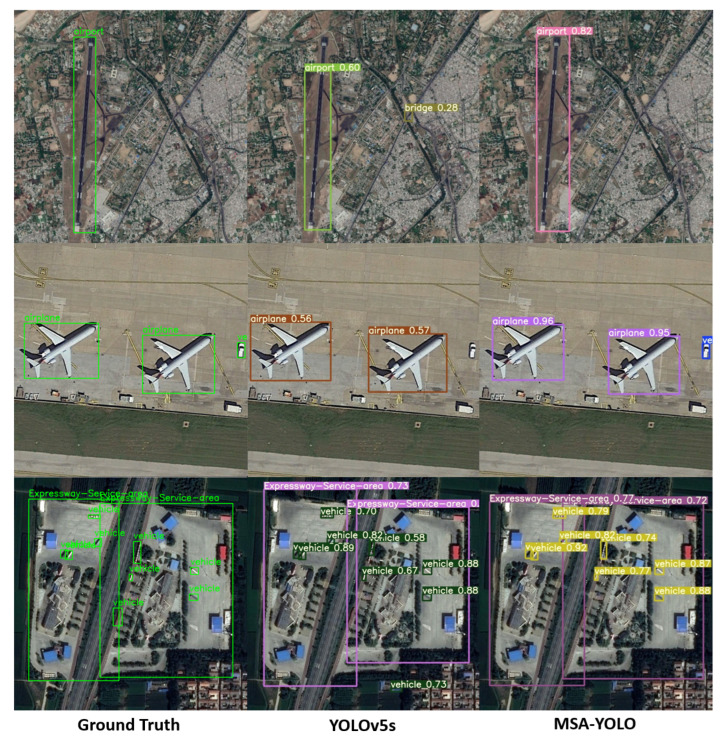
Detection results of YOLOv5s and MSA-YOLO.

Figure 14 shows the actual detection results of YOLOv5s and MSA-YOLO in remote sensing images. It can be seen that MSA-YOLO can effectively suppress the interference of background noise in detection. Compared with YOLOv5s, MSA-YOLO has a higher confidence score when detecting the same object and can also detect smaller objects, reducing the occurrence of missed detection and false detection. The detection results prove the effectiveness and superiority of the proposed model.

We compared MSA-YOLO with widely used advanced detectors in comparative experiments. Table 5 and Table 6 show the results of the comparative experiments on the DIOR dataset and HRRSD dataset. It can be seen from the experimental results that our proposed MSA-YOLO model has fewer parameters and achieves the best detection accuracy on both datasets. MSA-YOLO is more effective in detecting small objects, and we believe there are two main reasons for the enhancement: first, our MSCAM reduces the influence of irrelevant background information; thus small objects that are easy to lose information on receive more attention. Second, our improved loss function rebalances the regression weights for different samples, and small objects that are difficult to detect receive more regression weights. Overall, compared with other detectors, MSA-YOLO achieves a better balance between detection accuracy and model lightweighting.

## 5. Conclusions

Given the problems of complex background noise and significant differences in object scale in remote sensing image object detection, this article proposes an improved remote sensing image object detection model, MSA-YOLO, based on the YOLOv5s model, which is used for remote sensing object detection in complex scenes. First, we propose a new multi-scale fusion strip convolution attention mechanism MSCAM, which uses strip convolution to suppress complex background noise and fuse multi-scale features. In addition, we propose an improved feature fusion layer GS-PANet by introducing GSConv, which can better fuse semantic features and guarantee a lightweight model. Finally, we propose the Wise-Focal CIoU loss function, which reweights different samples according to their quality, flexibly adjusts the gradient contribution of different samples to the regression loss, and finally improves the regression effect of the bounding box. Experiments conducted on two large-scale remote sensing image datasets have shown that the performance of our proposed MSA-YOLO model is superior to other widely used object detectors in the remote sensing image object detection task. However, our current approach does not consider scenarios with dense object distribution and the presence of occlusion. In addition, there is still room for optimizing the lightweight design of our model compared to advanced lightweight models. In future work, we will consider methods to improve the accuracy of remote sensing image object detection by combining advanced models such as Vision Transformer or DETR. At the same time, we will explore methods such as knowledge distillation and model pruning to improve the deployability of the model further. 

## Figures and Tables

**Table 1:** Performance comparison using different design schemes of GS-PANet on the DIOR dataset.

Method	AP50	AP50:95	Params(M)
GS-PANet1	78.0	56.3	6.67
GS-PANet2	78.2	56.5	6.63
GS-PANet3	77.9	56.3	6.47

**Table 2:** Ablation results on different hyperparameters.

Method	${\mathrm{AP}}_{50}$	${\mathrm{AP}}_{75}$	${\mathrm{AP}}_{50:95}$
Wise-Focal CIoU γ=2	77.4	60.9	56.2
Wise-Focal CIoU γ=3	77.3	60.6	56.2
Wise-Focal CIoU γ=4	77.2	60.3	56.1
Wise-Focal CIoU γ=5	76.9	60.4	56.1
Wise-Focal CIoU γ=6	76.2	60.0	55.7

**Table 3:** Ablation experiments.

Method	MSCAM	GS-PANet	Wise-Focal CIoU Loss	${\mathrm{AP}}_{50}$	${\mathrm{AP}}_{50:95}$	Params (M)
YOLOv5s (Baseline)				77.7	55.7	7.11
1	✓			79.2	57.7	7.38
2		✓		78.2	56.5	6.63
3			✓	77.4	56.2	7.11
4		✓	✓	78.3	57.2	6.63
MSA-YOLO	✓	✓	✓	79.3	58.6	6.91

**Table 4:** Performance comparison of different attention mechanisms.

Attention Mechanism	${\mathrm{AP}}_{50}$	${\mathrm{AP}}_{75}$	${\mathrm{AP}}_{S}$	${\mathrm{AP}}_{M}$	${\mathrm{AP}}_{L}$	${\mathrm{AP}}_{50:95}$	Params (M)
No (Baseline)	77.7	60.4	11.4	38.4	67.5	55.7	7.11
SENet [28]	78.0	60.4	11.2	38.2	67.2	55.7	7.15
CBAM [30]	77.4	60.2	10.5	38.4	67.3	55.6	7.18
TAM [32]	78.4	60.5	10.8	38.6	67.7	56.1	7.11
CA [31]	78.6	61.2	12.1	39.3	56.4	67.9	7.22
Non-Local [33]	78.2	60.7	10.7	39.5	67.4	56.0	7.63
BRA [35]	78.3	60.6	11.5	38.6	67.6	56.2	8.16
MSCAM	79.2	62.7	11.8	39.5	69.7	57.7	7.38

**Table 5:** Performance comparison of different models on the DIOR dataset.

Model	${\mathrm{AP}}_{50}$	${\mathrm{AP}}_{75}$	${\mathrm{AP}}_{S}$	${\mathrm{AP}}_{M}$	${\mathrm{AP}}_{L}$	${\mathrm{AP}}_{50:95}$	Params (M)
Faster RCNN [3]	73.6	56.5	7.4	30.9	63.0	51.2	41.22
Cascade RCNN [4]	74.1	59.8	7.7	33.6	67.2	54.5	68.96
YOLOv3 [9]	75.7	60.6	10.1	36.5	67.6	55.5	62.67
YOLOv5s [11]	77.7	60.4	11.4	38.4	67.5	55.7	7.11
YOLOX-S [14]	76.5	58.2	10.6	37.6	65.3	53.9	8.96
YOLOv7-tiny [12]	78.9	62.3	10.1	39.0	69.6	57.6	6.06
MSA-YOLO (ours)	79.3	63.3	12.6	40.1	70.3	58.6	6.91

**Table 6:** Performance comparison of different models on the HRRSD dataset.

Model	${\mathrm{AP}}_{50}$	${\mathrm{AP}}_{75}$	${\mathrm{AP}}_{S}$	${\mathrm{AP}}_{M}$	${\mathrm{AP}}_{L}$	${\mathrm{AP}}_{50:95}$	Params (M)
Faster RCNN [3]	88.4	67.9	34.4	50.3	56.9	59.1	41.22
Cascade RCNN [4]	88.3	71.0	34.4	51.8	59.8	62.0	68.96
YOLOv3 [9]	89.5	68.9	36.9	51.4	59.5	61.3	62.67
YOLOv5s [11]	91.7	74.8	41.7	53.7	62.8	65.5	7.11
YOLOX-S [14]	91.4	73.2	42.8	54.2	62.0	64.8	8.96
YOLOv7-tiny [12]	91.5	73.3	41.3	53.3	62.3	64.7	6.06
MSA-YOLO (ours)	93.0	77.0	47.1	54.5	64.8	67.4	6.91

## Data Availability

The datasets used in this study are all public datasets.
